# Product Development and Consumer Evaluation of Beef–Cricket Hybrid Burgers

**DOI:** 10.1155/ijfo/5598812

**Published:** 2026-04-22

**Authors:** Leocardia Ranga, Francesco Noci, Maria Dermiki

**Affiliations:** ^1^ Department of Health and Nutritional Sciences, School of Health, Sport Sciences and Nutrition, Faculty of Science and Health, Atlantic Technological University, Sligo, Ireland; ^2^ Department of Sport, Exercise and Nutrition, School of Health, Sport Sciences and Nutrition, Faculty of Science and Health, Atlantic Technological University, Galway, Ireland

## Abstract

The need for sustainable protein alternatives has led to an exploration of edible insects as meat extenders. This study investigated the feasibility of reducing beef content in burgers by incorporating insect‐based ingredients and examined the effect on physicochemical, techno‐functional and sensory properties, as well as consumer acceptance and willingness to buy hybrid beef–insect products. Beef–insect hybrid burgers were developed by replacing 10%–40% of ground beef with a cricket‐based ingredient formulated to approximate the protein and functional properties of beef. The physicochemical properties (colour, texture, moisture and cooking loss) were tested, and consumer preference testing was conducted with 67 participants. Data were analysed using ANOVA, nonparametric tests, and multiple linear regression. Replacement of beef up to 40% did not significantly affect texture, moisture, or cooking loss compared with the control beef‐only burger. Colour differences were limited to the lightness (*L* ^∗^) of raw samples, with the samples with a 40% replacement level appearing darker. Consumer rankings showed a clear preference for higher beef content; however, no significant differences were observed between the control and burgers with 20% beef replacement, suggesting that moderate substitution can be achieved without reducing acceptance. Regression analysis revealed that environmental impact, cost and protein content jointly explained 31% of the variance in willingness to buy, with environmental impact and cost exerting the strongest negative effects. Overall, cricket‐based hybrid burgers demonstrated technological feasibility and partial consumer acceptance, particularly at modest substitution levels (≤ 20%). Broader adoption will depend on maintaining sensory appeal while communicating nutritional and environmental benefits effectively. These findings highlight the potential of insect‐based hybrid meats as a practical pathway towards more sustainable protein consumption in Western markets.

## 1. Introduction

Over the past decade, interest in entomophagy has grown in Western societies, largely driven by the need for sustainable protein alternatives [1]. This shift is mainly prompted by increasing global demand for animal protein, which exacerbates environmental challenges such as greenhouse gas emissions, land degradation and water scarcity [2, 3]. Current projections suggest that global per capita meat intake will continue rising, with an estimated increase of approximately half a kilogram per person per year by 2033 [4]. In response, insect‐based foods have begun to emerge in Western markets, though their introduction remains gradual and uneven [5].

Within the European Union (EU), where insects are regulated under a unified novel food framework [6], market exposure varies considerably among member states [5]. Products available across the broader EU online market vary widely in form and processing levels, from whole dried insects and insect‐based confectionery to ground flours and ready‐to‐eat meals where insects are no longer visually identifiable [7]. However, many of these products contain insect species not yet approved for consumption across all EU member states, creating regulatory and trade limitations [8]. Furthermore, online availability does not guarantee consumer exposure. Unlike physical retail environments, where consumers might encounter products passively, insect‐based foods sold online require intentional searching, limiting incidental discovery [9]. Given that consumer acceptance of insect‐based foods differs across countries [10], there remains a need to explore acceptance and introduce such products into physical retail markets, particularly in regions where they are not yet available [7].

While snack‐style insect products may help familiarise consumers with the idea of insects as food, they have limited potential as meat substitutes [11]. Conversely, insect‐enriched foods such as pasta could function as a meat alternative when combined with vegetarian sauces [12]. Yet, consumer studies consistently show that meat attachment negatively impacts the acceptance of insect‐based foods [13, 14]. To address this, plant‐based analogues have been designed to mimic the sensory and structural characteristics of meat [15, 16]. Recent innovations include combining plant and insect proteins to produce hybrid burgers and jerky [17–19]. Despite these advances, replicating meat’s complex sensory and textural attributes remains challenging, often leading to lower consumer acceptance [15, 20].

Rather than eliminating meat entirely, a gradual approach of retaining some while introducing insects may help bridge the gap between sustainability goals and consumer acceptance [21]. Previous research has examined the use of alternative proteins such as legumes and insects as meat extenders [16, 22–29]. Examples include hybrid beef burgers [16, 30] and pork frankfurters [31] incorporating *Tenebrio molitor* larva (mealworms), as well as beef burgers containing *Hermetia illucens* larva (black soldier fly) [32]. However, a persistent challenge across such studies has been aligning the physicochemical and techno‐functional properties (properties describing ingredient or product performance during processing and cooking) of reformulated products with those of conventional meat [23, 25, 31–36]. Moreover, a 2022 review highlighted that, although research on insects as meat extenders is expanding, studies on sensory acceptability remain limited [26], a gap that recent work has begun to address [33–35].

In Ireland, consumer exposure to insect‐based foods remains minimal. Although a hybrid burger containing mealworms and soy was briefly introduced by Lidl Ireland in 2023, it was not permanently stocked [37, 38]. As of the time this study was conducted (February 2025), no insect‐based products were available in Irish retail outlets. This limited exposure, combined with Ireland’s cultural and economic ties to livestock farming, particularly beef and sheep production, which sustain nearly 200,000 livelihoods [39, 40], creates a unique context for exploring hybrid meat strategies. Reducing, rather than eliminating, meat in widely consumed foods such as burgers [41] may therefore provide a more socially acceptable and economically viable path towards dietary change.

Among animal proteins, beef production has one of the highest environmental footprints, particularly in terms of greenhouse gas emissions, land use and water consumption [42–44]. Partial substitution of beef with insect‐derived ingredients could yield meaningful environmental benefits while preserving the sensory and nutritional characteristics valued by consumers.

Against this backdrop, the present study aimed to develop and evaluate beef–insect hybrid burgers formulated to reduce beef content while maintaining comparable protein levels and key functional properties (colour, texture, moisture and cooking loss). This approach partially aligns with that used in previous research by Hashempour–Baltork and colleagues when investigating the use of mycoprotein as a replacement for chicken in nuggets [45]. In the current study, burgers were selected as the carrier food because they are both familiar and popular among consumers in Ireland [41], providing a practical vehicle for protein substitution. Specifically, this study sought to the following:

1. Develop beef–insect hybrid burgers by partially replacing beef with an insect‐based ingredient designed to approximate the protein and techno‐functional properties of conventional beef.
2. Evaluate consumer acceptance of the hybrid burgers among a segment of consumers in Ireland, examining the effect of varying levels of beef replacement on sensory appeal and overall preference.
3. Identify consumer‐ and product‐related factors influencing acceptance and willingness to purchase beef–insect hybrid burgers among a segment of consumers in Ireland.

## 2. Materials and Methods

### 2.1. Materials

Insect flours used in this study included mealworm flour (from dried, ground *T. molitor* larvae) and cricket flour (from dried, ground *Acheta domesticus*), both purchased online from Catch‐your‐Bug (Schnürpflingen, Germany). The mealworm flour contained 55.1 g of protein/100 g, and cricket flour contained 66.3 g of protein/100 g. Ground beef (9.7% fat, 20.4 g protein/100 g), breadcrumbs, onions, seasoning and oil were sourced from a local supermarket (Tesco, Sligo, Ireland), while potato fibre was kindly supplied by Provil (Thessaloniki, Greece). A 3‐in‐1 Claspme burger patty maker was purchased online from Amazon (London, UK).

### 2.2. Burger Formulation and Preparation

**FIGURE 1:**
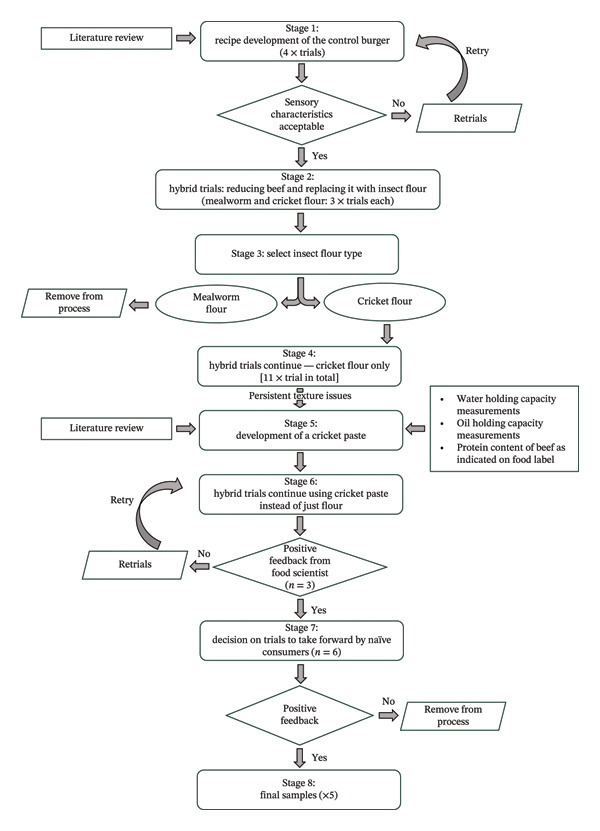
Schematic presentation of the product development process for the insect‐containing burgers.

The development of the burgers followed seven stages (see Figure 1). The process began with the formulation of a control beef burger recipe, adapted from a past study [16]. Four iterative trials were conducted to optimise sensory properties before finalising the control formulation. At each stage, continuation or modification of formulations was guided by informal sensory evaluations involving at least three food scientists, except in the final stage, which also included six regular consumers.

In the second stage, hybrid burger trials were initiated by progressively reducing beef content from the control formulation and substituting it with insect flour. Six trials were performed in total, three with yellow mealworm flour and three with cricket flour. These species were selected, as they were the only insect powders authorised and commercially available within the EU at the time of the study (February 2025). The use of flour was informed by prior consumer insights indicating higher acceptance of insect‐based products in which insects are not visually identifiable [7, 46].

Preliminary evaluations indicated superior sensory performance for cricket‐based samples compared to mealworm‐based samples, which exhibited a coarse texture and visible insect fragments. Cricket flour also contained a higher protein content (66.3 g/100 g) than mealworm flour (55.1 g/100 g), supporting its selection for subsequent trials. The maximum acceptable inclusion level was determined to be 10 g cricket flour per 100 g burger to avoid off‐flavours. Subsequent hybrid formulations therefore focused on reducing beef while maintaining protein equivalence without exceeding this inclusion level.

Persistent texture issues observed in these formulations prompted the development of a cricket paste (a hydrated formulation of cricket flour) to improve moisture retention and cohesiveness. This approach was informed by the average water content of 10% fat ground beef (> 60%) [46, 47] and the measured water‐ and oil‐holding capacities of cricket flour (2.02 ± 0.10 g/g and 1.70 ± 0.04 g/g, respectively) using the method described elsewhere [48]. The paste formulation (ingredients/100 g paste: 30.5 g cricket flour, 5.9 g potato fibre, 61.0 g water and 2.6 g oil) was designed to approximate the protein content of the beef it replaced. Table 1 summarises the calculated nutritional composition of the ground beef and cricket paste based on food label values.

**TABLE 1:** Nutritional comparison of the ground beef and cricket paste (which were used as ingredients in the burgers developed in this study), calculated based on the nutritional information provided on the food label of each ingredient used.

Nutritional information per 100 g		
Component	Ground beef	Cricket paste
Energy (kJ/kcal)	704/168	694/166
Fat (g)	9.7	9.0
Of which saturates (g)	4.5	2.9
Carbohydrate (g)	0	0.2
Of which sugars (g)	0	0.1
Fibre (g)	0	5.0
Protein (g)	20.4	20.2
Salt (g)	0.2	0.2

Hybrid burger trials using cricket paste were then conducted to refine texture and functional properties. A final screening with six consumers was used to identify formulations for sensory testing. Seven samples (control plus hybrids with 5%, 10%, 15%, 20%, 30% and 40% beef reduction) were evaluated monadically using five‐point scales for overall liking, appearance, flavour and texture (see Table S1 in the supporting information for the hedonic scores). Following a group discussion, five samples were selected for the main sensory evaluation (to allow all samples to be evaluated within a single session without inducing sensory fatigue): The control and hybrids with 10%, 20%, 30% and 40% beef replaced. The final ingredient compositions for each of these samples are presented in Table 2.

**TABLE 2:** Ingredients used per 100 g of each burger.

Ingredient^1^	Type of burger sample				
	Control	Beef–insect hybrid burger with the following percentage of beef replaced:			
		10%	20%	30%	40%
Ground beef	77.0	69.3	61.6	53.9	46.2
Cricket paste	0.0	7.7	15.4	23.1	30.8
Breadcrumbs	8.0	8.0	8.0	8.0	8.0
Tomato purée	2.0	2.0	2.0	2.0	2.0
Brown onion^2^	5.0	5.0	5.0	5.0	5.0
Garlic powder	0.8	0.8	0.8	0.8	0.8
Black pepper	0.1	0.1	0.1	0.1	0.1
BBQ seasoning	0.4	0.4	0.4	0.4	0.4
Worcestershire sauce	5.0	5.0	5.0	5.0	5.0
Yeast extract	1.7	1.7	1.7	1.7	1.7

^1^All values are in grams per 100 g of the sample.

^2^Finely chopped.

All sample handling and preparation followed good hygiene practices and Hazard Analysis and Critical Control Point (HACCP) principles in accordance with Regulation (EC) No 852/2004 [49]. All ingredients (which were commercially sourced) were gently mixed to prevent overworking [50] and then portioned into 50 g patties (7.3 cm diameter) using a burger press and stored below −18°C. Patties were thawed under refrigeration (< 5°C) prior to cooking in a Rational combi oven (Landsberg am Lech, Germany) at 180°C for 15 min, ensuring a core temperature of ≥ 75°C [51]. Cooked samples were hot held at 65°C with 60% humidity to maintain texture and moisture until sensory evaluation.

### 2.3. Assessment of Physicochemical and Techno‐Functional Properties

#### 2.3.1. Colour Measurement

The colour of the burgers was evaluated using a Lovibond LC‐100 colorimeter (The Tintometer Ltd., Amesbury, UK) in the CIE *L* *a* *b* ^∗^ colour space. Colour measurements were conducted on both raw and cooked samples to capture any changes resulting from cooking. For the cooked samples, measurements were taken separately from the interior and exterior surfaces to distinguish colour differences caused by exposure to direct heat and surface browning. Calibration of the colorimeter was carried out at the beginning of each measurement session using a built‐in white background. For each parameter (*L* ^∗^, *a* ^∗^ and *b* ^∗^), three separate readings (*n* = 3) were taken at different points on each sample to ensure accuracy.

#### 2.3.2. Texture Analysis

Texture attributes, such as firmness and toughness, were measured using a TA. XT texture analyser (Stable Micro Systems, Surrey, UK) equipped with a Warner–Bratzler blade and slotted insert. Samples were positioned perpendicular to the slot during testing, and the test settings were pre‐test speed = 2.0 mm/s, test speed = 2.0 mm/s, post‐test speed = 10.0 mm/s, return distance = 30 mm, trigger force = 20 g, contact force = 40 g, and data acquisition rate = 200 pps. Firmness was recorded as the peak cutting force (N), while toughness was calculated as the area under the force–time curve (N.s). Output forces in gram‐force were converted to SI units using the conversion factor 1 g = 0.00980665 N. Each measurement was repeated three times per sample (*n* = 3).

#### 2.3.3. Moisture Content

Moisture content was determined using an OHAUS MB23 moisture balance (OHAUS, Zurich, Switzerland). A consistent sample weight of 1.22 g was used, with three replicates per sample (*n* = 3). The balance temperature was set at 100°C for all tests.

#### 2.3.4. Cooking Loss

Cooking loss was assessed by recording the weight of each burger sample (*n* = 3) before and after cooking. The percentage cooking loss was then calculated using the following equation:

(1) cooking loss %=weight of raw sample−weight of cooked sampleweight of raw sample×100.

Moisture content and cooking loss were used to assess water retention behaviour in the burger samples.

### 2.4. Sampling and Sensory Evaluation Procedure

A total of 67 adult participants were recruited through multiple channels, including university mailing lists, social media platforms (X, WhatsApp and Workvivo), and posters placed in the educational institution, cafeterias and volunteer centres in the Sligo area. Recruitment targeted a sample size between 50 and 100, as recommended for preference ranking tests [52]. Participants were informed of the study details and screening criteria prior to enrolment. Exclusion criteria included being under 18 years of age, having food allergies, dust mite allergies, or medical conditions affecting food intake.

The study received ethical approval from the Institute Research Ethics Committee of Atlantic Technological University, Sligo, Ireland (Ref. No. 2023032). All participants provided written informed consent before participation. Sensory evaluations were conducted in individual sensory booths under controlled lighting using RedJade Sensory Software (RedJade Sensory Solutions, LLC, Martinez, CA, USA), following ISO 11136:2014 standards for consumer sensory testing [53].

Before tasting, participants completed a presensory questionnaire designed to collect demographic and behavioural information, including age, gender, whether they were working or studying in a food‐related field, frequency of beef burger consumption and prior experience with insect‐based foods. They were also asked to indicate their level of agreement with six items from the Domain Specific Innovativeness Scale [54], adapted for novel foods [55] and nine statements assessing beliefs and attitudes towards entomophagy [56, 57]. All responses were recorded on a seven‐point Likert scale ranging from *strongly disagree* to *strongly agree*.

Following completion of the questionnaire, participants proceeded to the sensory evaluation. Each participant received five burgers, one control and four beef–cricket hybrid formulations with 10%, 20%, 30% and 40% reductions in beef content. Samples were labelled with unique three‐digit codes and presented in a randomised order [52]. Each participant received one‐quarter of each burger sample and was instructed to assess and taste them sequentially from left to right and then rank them from most to least preferred. Crackers and water were provided to cleanse the palate between samples.

After ranking the samples, participants were asked an open‐ended question to describe the factors that influenced their preferences. They were then asked whether they would change their ranking if they knew that the sample they preferred most contained insect ingredients (yes/no).

Finally, participants completed a rating‐based conjoint task [58] to assess their hypothetical willingness to purchase their most preferred sample, assuming it was a beef–insect hybrid burger. Willingness to buy was rated on a nine‐point scale ranging from *extremely unwilling* to *extremely willing*. The conjoint design incorporated three product attributes, namely, environmental impact, protein content and cost, each defined relative to a traditional beef‐only burger at two levels (high and low), resulting in eight unique factorial combinations (2 × 2 × 2). A ninth combination, representing a hybrid burger identical to the beef‐only burger across all three attributes, was included as a baseline (Table 3).

**TABLE 3:** Total attribute combinations used for environmental impact, protein content and cost at varying levels (low, high or same) relative to a beef‐only burger.

Combination	Environmental impact	Protein content	Cost
1	Low	Low	Low
2	Low	Low	High
3	Low	High	Low
4	Low	High	High
5	High	Low	Low
6	High	Low	High
7	High	High	High
8	High	High	Low
9	Same	Same	Same

### 2.5. Data Analysis

Statistical analyses were performed using SPSS Statistics software (IBM, Version 29.0.1). For Likert‐type items measuring agreement, responses were coded on a seven‐point scale (1 = *strongly disagree* to 7 = *strongly agree*), while willingness‐to‐buy scores were coded on a nine‐point scale (1 = *extremely unwilling* to 9 = *extremely willing*).

The nutritional information of each burger formulation was calculated based on the nutritional values of the individual ingredients, as provided on their respective food labels. Differences in physicochemical and techno‐functional parameters between burgers were evaluated using one‐way analysis of variance (ANOVA).

Participant characteristics were analysed using descriptive statistics. Differences in participants’ preference rankings across burgers were analysed using the Friedman test. Where significant effects were found, post hoc pairwise comparisons were performed using Bonferroni correction to identify which sample pairs differed significantly. To further explore consumer‐ and product‐related factors influencing both preference rankings and willingness to buy the most preferred sample (hypothetically, a beef–insect hybrid burger), a series of statistical tests were applied (see Table 4). Due to unbalanced categories, gender effects included only two categories (male and female), while all categories were retained for the rest of the analysis. While the internal consistency of the Domain Specific Innovativeness Scale was acceptable (Cronbach’s *α* = 0.79), individual items were analysed separately to capture the influence of distinct aspects of innovativeness on product acceptance since a high Cronbach’s alpha does not necessarily indicate unidimensionality [59]. Significance for all statistical tests was set at *p* < 0.05.

**TABLE 4:** Overview of statistical tests used to analyse the effect of consumer and product‐related factors on preference rankings and willingness to buy.

Effect of	On	Statistical test used
Gender, working or studying in a food‐related field, knowing that the most preferred burger contains insects	Preference ranking of beef–insect hybrid burgers	Mann–Whitney U
Frequency of beef burger consumption, previous consumption of insect‐based foods		Kruskal–Wallis H
Age, level of agreement with items from the Domain Specific Innovativeness Scale and statements on beliefs and attitudes towards entomophagy (respectively)		Spearman’s correlation

Gender, working or studying in a food‐related field, knowing that the most preferred burger contains insects	Willingness to buy the most preferred product (hypothetically, a beef–insect hybrid burger)	Mann–Whitney U
Frequency of beef burger consumption, previous consumption of insect‐based foods		Kruskal–Wallis H
Age, level of agreement with items from the Domain Specific Innovativeness Scale and statements on beliefs and attitudes towards entomophagy (respectively), and preference ranking of beef–insect hybrid burgers		Spearman’s correlation
A combination of environmental impact, protein content and costs		Friedman
Environmental impact, protein content and costs (individually)		Multiple linear regression

The conjoint task assessing willingness to buy was analysed using a full factorial design comprising eight combinations of three product attributes (environmental impact and protein content and cost), each tested at two levels (low and high), with an additional baseline combination representing a hybrid burger identical to the conventional beef burger. A Friedman test was used to assess overall differences in willingness‐to‐buy scores across the nine combinations. To examine the effect of each individual attribute, a multiple linear regression model was applied using the eight factorial combinations. The dependent variable was willingness to buy (*Y*), and the independent variables, environmental impact (*X*_1_), protein content (*X*_2_) and cost (*X*_3_), were coded as binary dummy variables (0 = low, 1 = high):

(2) Y=β0+β1X1+β2X2+β3X3+ε,

where *β*_0_ represents the intercept and *ε* the model error.

Participant responses to the open‐ended question regarding the factors influencing their sample rankings were analysed using content analysis. The frequency with which each factor was mentioned for each sample was recorded. In addition, mentions under each factor were categorised as either positive or negative and tallied accordingly for each sample.

## 3. Results

### 3.1. Characteristics of the Burgers

#### 3.1.1. Visual Appearance

**FIGURE 2:**
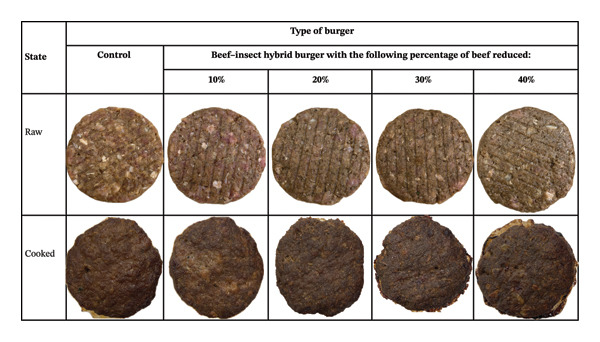
Photographic images of the burgers in raw and cooked states.

Figure 2 shows the final burgers in their raw and cooked state, which were used in the affective sensory evaluation.

#### 3.1.2. Nutritional Information and Ingredient Cost

The calculated nutritional information and ingredient cost per 100 g of each burger sample are summarised in Table 5. All hybrid burgers maintained a comparable protein content (17.4 g/100 g) to the control, while fibre content increased proportionally with the inclusion of cricket paste. Fat and energy values remained comparable across all formulations. Ingredient cost increased with higher levels of beef substitution due to the higher cost of cricket flour.

**TABLE 5:** Nutritional information calculated based on the food label of each ingredient and total ingredient cost per 100 g of burger.

Nutritional component	Type of burger sample				
	Control	Beef–insect hybrid burger with the following percentage of beef reduced:			
		10%	20%	30%	40%
Energy (kJ/)	689	688	688	687	686
Energy (kcal)	164	164	164	164	163
Fat (g)	7.6	7.6	7.5	7.5	7.4
Of which saturates (g)	3.5	3.4	3.3	3.2	3.0
Carbohydrate (g)	6.4	6.4	6.4	6.5	6.5
Of which sugars (g)	1.9	1.9	1.9	1.9	1.9
Fibre (g)	0.4	0.8	1.2	1.6	2.0
Protein (g)	17.4	17.4	17.4	17.4	17.4
Salt (g)	0.61	0.61	0.60	0.60	0.59

Total ingredient cost	€0.74	€0.96	€1.18	€1.40	€1.63

#### 3.1.3. Physicochemical and Techno‐Functional Properties

The results of instrumental analyses are summarised in Table 6. One‐way ANOVA indicated no significant differences (*p* > 0.05) among samples for most parameters, including texture (firmness and toughness), moisture content and cooking loss. The only significant difference observed was in the lightness (*L* ^∗^) value of the raw samples (*F* (4, 10) = 4.018, *p* = 0.034). A follow‐up Tukey HSD post hoc test revealed that the significant difference in the *L* ^∗^ values was between the control and the beef–insect hybrid burger with a 40% beef reduction. Specifically, there was a statistically significant decrease (*p* = 0.040) in the *L* ^∗^ value from 47.97 ± 3.219 in the control to 40.97 ± 1.904 in the 40% beef reduction sample—a difference of 7.0 units (95% CI, 0.3 to 13.7). The detailed values for all burgers are presented in Table 6.

**TABLE 6:** Physicochemical and techno‐functional properties of the burgers.

Physicochemical and techno‐functional properties	Type of burger					Significance (ANOVA)
	Control	Beef–insect hybrid burgers with the following percentage of beef reduced				
		10%	20%	30%	40%	
	Mean ± SD					*p*‐values
Colour: raw						
*L* ^∗^	47.97 ± 3.219^a^	43.80 ± 3.740^a,b^	41.87 ± 1.704^a,b^	41.33 ± 0.473^a,b^	40.97 ± 1.904^b^	**0.034**
*a* ^∗^	11.33 ± 2.178^a^	10.80 ± 0.755^a^	11.93 ± 1.124^a^	9.67 ± 0.493^a^	10.00 ± 1.400^a^	0.278
*b* ^∗^	23.37 ± 1.026^a^	22.10 ± 0.700^a^	21.33 ± 1.739^a^	20.80 ± 0.529^a^	20.40 ± 1.819^a^	0.106
Colour: cooked (interior)						
*L* ^∗^	35.37 ± 1.097^a^	43.23 ± 0.961^a^	37.00 ± 10.916^a^	39.57 ± 1.89^a^	38.57 ± 3.062^a^	0.458
*a* ^∗^	8.10 ± 0.625^a^	8.93 ± 1.206^a^	8.33 ± 0.351^a^	8.60 ± 0.100^a^	8.90 ± 1.652^a^	0.795
*b* ^∗^	17.97 ± 1.582^a^	20.80 ± 1.114^a^	28.77 ± 13.885^a^	19.47 ± 0.987^a^	19.93 ± 0.737^a^	0.312
Colour: cooked (exterior)						
*L* ^∗^	28.07 ± 0.569^a^	26.47 ± 3.017^a^	18.77 ± 9.205^a^	24.70 ± 0.721^a^	25.87 ± 2.967^a^	0.196
*a* ^∗^	10.83 ± 0.950^a^	10.77 ± 2.301^a^	8.93 ± 1.380^a^	9.63 ± 0.404^a^	9.20 ± 0.794^a^	0.330
*b* ^∗^	10.77 ± 3.450^a^	10.27 ± 1.320^a^	11.50 ± 8.058^a^	8.30 ± 0.625^a^	8.23 ± 1.888^a^	0.806
Texture: firmness (N)	9.39 ± 3.33^a^	10.08 ± 3.18^a^	8.24 ± 0.99^a^	9.78 ± 1.87^a^	7.76 ± 2.87^a^	0.774
Texture: toughness (N.s)	28.82 ± 4.14^a^	37.12 ± 8.76^a^	29.37 ± 4.05^a^	43.18 ± 13.03^a^	23.88 ± 5.35^a^	0.083
Moisture content (%)	40.97 ± 4.636^a^	46.37 ± 3.988^a^	42.20 ± 7.475^a^	39.53 ± 8.619^a^	34.00 ± 12.300^a^	0.476
Cooking loss (%)	29.53 ± 2.157^a^	30.67 ± 4.619^a^	27.70 ± 0.520^a^	28.67 ± 1.155^a^	28.20 ± 0.346^a^	0.584

*Note:* In bold—statistically significant value (only observed for the *L* ^∗^ colour values of the raw burgers); the same superscripts within each row indicate no significant differences between samples for that attribute.

### 3.2. Profile of the Participants

The mean age of participants was 28 years (SD = 11.4; range 18–61), with a near equal distribution of male and female participants, representing 44.8% and 53.7% of the total sample, respectively. Approximately 37% worked or studied in a food‐related field. Most participants consumed beef burgers occasionally (1–3 times per month), and 40% reported never having consumed insect‐based foods, while 28% had done so more than once (see Table 7).

**TABLE 7:** Characteristics of the participants (*N* = 67).

Profile	Participants (*N* = 67)
Age:	
Mean ± SD (min; max)	28.0 ± 11.4 (18; 61)
Gender:	
Male	44.8%
Female	**53.7%**
Other	1.5%
Prefer not to say	0.0%
Working or studying in a food‐related field:	
Yes	37.3%
No	**62.7%**
Frequency of beef burger consumption:	
Never	3.0%
Rarely (less than once a month)	31.3%
A few times a month (1–3 times per month)	**53.7%**
Once or twice a week	11.9%
Several times a week (3–6 times per week)	0.0%
Every day	0.0%
Previous consumption of insect‐based foods:	
No, never	**40.3%**
Unsure	13.4%
Yes, once	17.9%
Yes, more than once	28.4%

*Note:* min = minimum age, max = maximum age. In bold: group with the highest percentage within each variable.

### 3.3. Preference Ranking of the Samples by Participants

The ranking of the burgers by participants, based on preference, is presented in Table 8. A Friedman test indicated that the differences in rankings were statistically significant (*p* < 0.001). Overall, the control burger was the most preferred, followed by the beef–insect hybrid burgers in descending order of beef content, that is, those with 10%, 20%, 30% and, finally, 40% beef reduction. However, follow‐up post hoc pairwise comparisons revealed no significant differences (*p* > 0.05) in the preference rankings between the control burger and the beef–insect hybrid burgers with 10% and 20% beef reduction.

**TABLE 8:** Ranking of the burgers by participants in order of preference.

Type of burger	Friedman test			
	Mean rank	Ranking^1^	Significance	
			Overall *p*‐value^2^	Post hoc^3^
Control	1.99	1	**< 0.001**	a
Beef–insect hybrid: 10% reduced beef	2.40	2		a
Beef–insect hybrid: 20% reduced beef	2.73	3		a
Beef–insect hybrid: 30% reduced beef	3.54	4		b
Beef–insect hybrid: 40% reduced beef	4.34	5		c

*Note:* In bold—statistically significant value.

^1^Ranked from *most preferred* (1) to *least preferred* (5).

^2^ *p*‐value from the Friedman test prior to post hoc comparisons.

^3^Summary of post hoc test results—different letters indicate statistically significant differences between samples.

### 3.4. Factors Affecting the Preference Ranking of the Samples

#### 3.4.1. Consumer‐Related Factors

Overall, participants’ age, gender, frequency of beef burger consumption and awareness that their most preferred burger contained insects had no significant effect on their sample preferences (*p* > 0.05). However, working or studying in a food‐related field negatively influenced the ranking of the beef–insect hybrid burger with 40% beef reduction (*U* = 372.0, *p* = 0.026).

Similarly, previous consumption of insect‐based foods had a significant effect on the ranking of the control burger (*H* (3) = 7.991, *p* = 0.046) and the beef–insect hybrid burger with a 30% beef reduction (*H* (3) = 8.735, *p* = 0.033). For the control burger, participants who had consumed insect‐based foods more than once ranked it significantly lower (i.e., higher preference) than those who had only tried it once. In contrast, for the 30% beef‐reduced burger, participants who reported never having consumed insect‐based foods gave it a significantly lower ranking than those who were unsure if they had consumed such foods.

Overall, there were no significant correlations between innovativeness scores and sample rankings (*p* > 0.05), indicating that participants’ self‐reported food innovativeness did not influence their preferences. On the other hand, there were significant correlations between participants’ agreement with two entomophagy‐related beliefs and attitudes statements and their preference rankings. Agreement with the statements “Eating insects is disgusting” and “It is not natural for humans to eat insects” was positively correlated with the ranking of the beef–insect hybrid burger with a 30% beef reduction (*r* (65) = 0.369, *p* = 0.002 and *r* (65) = 0.295, *p* = 0.015, respectively). Greater agreement with these statements was associated with reduced preference for this burger. All other correlations were not statistically significant (*p* > 0.05).

#### 3.4.2. Product‐Related Factors

Qualitative analysis identified several product‐related factors that influenced participants’ rankings of the burgers. These factors are primarily related to the appearance, smell, basic taste sensations, flavour perception (including aroma‐related notes) and texture. Table 9 presents the frequency with which each factor was mentioned per sample, along with whether the mention was positive or negative.

**TABLE 9:** Frequency of positive (+) and negative (−) comments on product characteristics by burger sample.

Factor	Frequency mentioned										Total
	Control burger		Beef–insect hybrid burger with the following percentage of beef reduced:								
			10%		20%		30%		40%		
	+	−	+	−	+	−	+	−	+	−	
Appearance	2	0	1	0	0	0	0	0	0	1	4
Smell	3	0	0	0	0	0	0	0	0	0	3
Taste	26	0	17	2	10	2	2	8	0	12	79
Flavour	6	0	4	0	2	0	1	3	1	6	23
Texture	16	1	11	0	8	2	3	6	1	7	55

Total	53	1	33	2	20	4	6	17	2	26	

Overall, the control burger received the highest number of positive comments (*n* = 53), followed by the 10% beef reduced hybrid (*n* = 33), 20% (*n* = 20), 30% (*n* = 6) and 40% (*n* = 2) beef reduced hybrids. The control, 10% and 20% hybrid burgers received very few negative comments from participants (1, 2 and 4, respectively), whereas the 30% and 40% hybrids received a considerably higher number of negative remarks (17 and 26, respectively).

Across all samples, taste was the most frequently mentioned factor (*n* = 79), followed by texture (*n* = 55), flavour (*n* = 23), appearance (*n* = 4) and smell (*n* = 3).

Of the four comments related to appearance, three were positive (two for the control and one for the 10% hybrid) and one was negative, describing the 40% hybrid as having a “dark” and “less appealing” colour. All smell‐related comments (*n* = 3) were positive and exclusively related to the control burger; no other samples received comments on aroma.

In terms of taste, the control burger received no negative feedback, while the 40% beef reduced hybrid received no positive feedback. This sample was often described as having a “weird”, “strange” and “bitter” taste, particularly in comparison with other hybrids.

No negative flavour‐related comments were made about the control, 10% or 20% beef reduced hybrid burgers, whereas the 30% and 40% hybrids received only 3 and 6 negative comments, respectively. Texture comments were mostly positive for the control and 20% hybrid samples. Notably, only the 10% hybrid burger received no negative texture‐related feedback, while others received a few.

### 3.5. Factors Affecting Willingness to Buy

#### 3.5.1. Consumer‐Related Factors

Age and previous insect consumption significantly affected willingness to buy specific hybrid variants. Older participants and those who had previously eaten insects more than once were more willing to purchase the hybrid burger with lower environmental impact, lower protein content and higher cost (*r* = 0.290, *p* = 0.017; *H* (3) = 8.084, *p* = 0.044).

Innovativeness was also associated with willingness to buy under certain attribute conditions. Agreement with “I will buy new, different, or innovative foods even if I haven’t tasted them beforehand” was positively correlated with willingness to buy the hybrid burger with lower environmental impact, higher protein content and lower cost (*r* = 0.335, *p* = 0.006). However, agreement with “Compared to my friends, I buy a lot of new, different or innovative foods”, was significantly negatively correlated with willingness to buy the same hybrid burger (*r* (65) = −0.248, *p* = 0.043).

Beliefs about entomophagy significantly predicted willingness to buy. Agreement with “insects are highly nutritious” and “eating insects is good for the environment” was positively correlated (*r* = 0.448, *p* < 0.001; *r* = 0.264, *p* = 0.031), while agreement with “eating insects is disgusting” and “it is not natural for humans to eat insects” was negatively correlated (*r* = −0.430, *p* < 0.001; *r* = −0.397, *p* < 0.001).

#### 3.5.2. Product‐Related Factors

The combination of product attributes (environmental impact and protein content and cost) significantly influenced willingness to buy (Friedman *p* < 0.001). Participants were most willing to purchase the hybrid burger that was lower in environmental impact, higher in protein content and lower in cost compared with a conventional beef burger (mean = 7.82 ± 1.37).

A multiple linear regression (*F* (3, 532) = 82.510, *p* < 0.001, *R* ^2^ = 0.314) confirmed that all three attributes were significant predictors of willingness to buy (see Table 10). Environmental impact (*B* = −1.903, *p* < 0.001) and cost (*B* = −1.888, *p* < 0.001) had negative effects, while protein content (*B* = 0.978, *p* < 0.001) had a positive effect. Environmental impact was the strongest predictor, followed closely by cost.

**TABLE 10:** Results of the multiple linear regression predicting willingness to buy a hybrid burger.

Willingness to buy predictor	*B*	95% CI for *B*		SE *B*	Β	*R* ^2^	Δ*R* ^2^
		LL	UL				
Model						0.32	0.31^∗∗∗^
Constant	6.34^∗∗∗^	5.99	6.70	0.18			
Environmental impact	−1.903^∗∗∗^	−2.26	−1.55	0.18	−0.38^∗∗∗^		
Protein content	0.978^∗∗∗^	0.621	1.334	0.18	0.19^∗∗∗^		
Cost	−1.888^∗∗∗^	−2.24	−1.53	0.18	−0.37^∗∗∗^		

*Note: B* = unstandardised regression coefficient; *SE B* = standard error of the coefficient; *β* = standardised coefficient; *R* ^2^ = coefficient of determination; Δ*R* ^2^ = adjusted *R* ^2^.

Abbreviations: CI = confidence interval, LL = lower limit, UL = upper limit.

^∗∗∗^ *p* < 0.001.

## 4. Discussion

This study investigated the impact of partially substituting beef with a cricket‐based ingredient on the physicochemical, techno‐functional, and sensory properties of beef burgers, as well as on consumer acceptance and willingness to buy hybrid beef–insect products.

The physicochemical and techno‐functional analyses indicated that incorporating cricket paste at substitution levels up to 40% did not significantly alter most quality parameters compared with the control. No significant differences were observed in texture, moisture content, or cooking loss, while colour differences were limited to the lightness (*L* ^∗^) of raw samples, where the 40% hybrid burger appeared darker. These findings are consistent with those of Rocchetti et al. [29], who reported that cricket‐enriched burgers were darker in raw form but exhibited no significant differences after cooking. The use of hydrated cricket flour in this study likely contributed to the maintenance of comparable functional properties by improving the binding and moisture retention of the mixture.

Previous studies using dry insect flours have often reported negative effects on texture and juiciness [32], supporting the current approach of incorporating hydrated cricket flour rather than dry powder. This suggests that the paste format may mitigate some of the undesirable structural and visual changes commonly associated with insect ingredients, allowing for higher substitution levels while maintaining acceptable product characteristics. Nonetheless, the present study demonstrates that maintaining comparable functional properties between hybrid and conventional burgers does not necessarily translate to consumer acceptance, underscoring the complexity of developing insect‐based food products that are both technically viable and acceptable to consumers.

Consumers generally preferred burgers with higher beef content, a pattern consistent with previous studies on hybrid meat products [16, 29]. However, no significant differences were observed between the control and the hybrids containing 10% or 20% beef reduction, indicating that modest inclusion of cricket paste can be achieved without negatively affecting consumer liking. A significant decline in preference at higher substitution levels (≥ 30%) suggests a perceptual threshold beyond which either sensory changes or psychological barriers reduce acceptance. Qualitative feedback supported this interpretation, as participants described higher‐substitution hybrids as having less appealing taste, texture and appearance.

Recent studies further support the existence of sensory acceptance thresholds when insects are used as meat extenders, although these thresholds appear to depend on both the insect ingredient and the product matrix. Pasqualin Cavalheiro et al. [34] reported that beef burgers with 5% replacement of beef with cricket flour achieved sensory scores comparable to beef‐only controls, while higher replacement levels (≥ 7.5%) resulted in significantly reduced liking. Similarly, Carvalho et al. [35] found that replacing 25% of pork in cooked ham with 10% lesser mealworm, or a blend of lesser and yellow mealworm (5% each), yielded sensory properties comparable to conventional ham. Taking the present findings into consideration as well, these studies suggest that acceptable substitution levels are highly product‐specific and influenced by formulation strategy, insect species and processing format, reinforcing the need to evaluate hybrid meat products within their intended application rather than through direct numerical comparison across studies.

**FIGURE 3:**
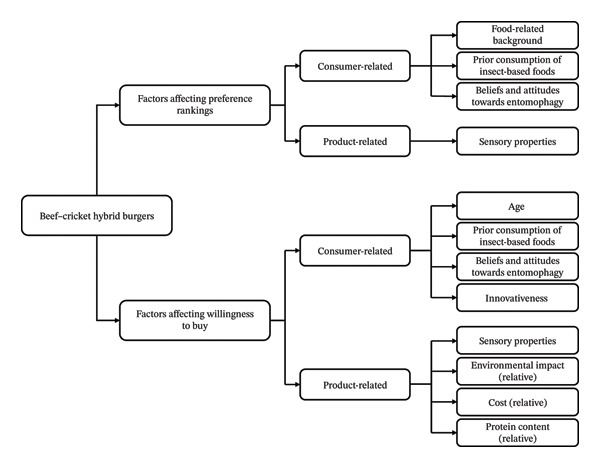
Summary of the consumer and product‐related factors affecting preference rankings of the burgers and willingness to buy the most preferred burger (hypothetically a beef–insect hybrid burger) relative to the beef burger.

A range of consumer‐ and product‐related factors further influenced these preferences (see Figure 3). Among the eight consumer‐related factors examined, three (food‐related background, previous insect‐based food consumption and entomophagy‐related beliefs and attitudes) influenced preference rankings. Four factors (age, previous insect‐based food consumption and innovativeness and entomophagy‐related beliefs and attitudes) affected willingness to buy.

At the product level, environmental impact, protein content and cost were all significant predictors of purchase intent, jointly explaining 31.4% of its variance. Lower environmental impact and higher protein content increased willingness to buy, whereas higher cost reduced it. This suggests that while environmental sustainability is a compelling factor, consumers are highly responsive to pricing and nutritional value, emphasising the importance of multi‐attribute alignment in product design, as was also found in past research [60]. While higher ingredient costs are currently a barrier for insect‐based products, these may be mitigated through industrial scaling and automation, supply chain optimisation, or targeted policy support as the sector matures [61]. Moreover, as only minimal information was provided in the present study, future work should examine whether positive framing of environmental and nutritional benefits further enhances consumer acceptance, as suggested by previous research on insect‐based foods [1, 62].

While participants’ age did not significantly affect their preference rankings of the burgers, it did affect their willingness to buy a beef–insect hybrid burger. Older participants were more willing to buy a hybrid with lower environmental impact, even when these came with trade‐offs, such as lower protein content and higher cost compared to conventional beef burgers. Previous research on the influence of age on insect‐based food acceptance has yielded mixed results [1, 63]. However, it was found elsewhere that older consumers in France and Ireland were more willing to consume insect‐based products under specific conditions, related to branding and packaging, which are usually attributed to higher costs [64–66]. In the present study it is possible that older consumers associated higher cost with other quality product attributes in this case.

Participants with a food‐related background ranked the 40% beef‐reduced hybrid burger significantly lower compared to those without such a background, indicating a lower preference. This effect was not observed for the control or other hybrid samples. Previous studies have mostly investigated the effect of level of education as opposed to the field of education or employment [1, 63]. A possible explanation of the result observed in the current study is that individuals with food‐related training may have heightened sensitivity to changes in sensory characteristics such as taste, particularly when the proportion of insect‐based content becomes more substantial. Thus, while they may not oppose the idea of consuming insect‐based foods [7], their lower ranking of the most heavily modified sample may reflect more critical assessments of its sensory acceptability rather than a general unwillingness to adopt such products.

Previous consumption of insect‐based foods significantly influenced both preference rankings and willingness to buy. Participants who had never consumed insect‐based foods ranked the 30% beef‐reduced hybrid burger lower (indicating higher preference) than those uncertain about their prior consumption. These findings suggest that complete lack of familiarity may not necessarily reduce preference for moderately reformulated hybrid products, whereas uncertainty about prior exposure may reflect greater hesitancy. In addition, those with repeated insect consumption were more willing to purchase a hybrid burger with lower environmental impact, despite its lower protein content and higher cost. This may indicate that repeated exposure fosters openness to alternative proteins and a willingness to accept trade‐offs for environmental benefits.

Participants’ innovativeness scores significantly correlated with their willingness to buy specific hybrid variants, but not with their preference rankings. This is unsurprising, as the innovativeness scale primarily measures openness to purchasing novel foods rather than sensory preferences. Participants willing to buy without prior tasting were more inclined to purchase hybrids with lower environmental impact, higher protein content and lower costs. This aligns with the regression results as well as what was reported in past studies as factors motivating consumers to try insect‐based foods [60, 67, 68]. However, those who frequently purchased new or innovative foods were less willing, suggesting that novelty alone may not sustain long‐term adoption. Past research indicates that sustainability benefits alone may not convince consumers to integrate insect‐based foods into their regular diets; instead, a combination of sensory appeal, availability and affordability is more persuasive [60, 69–71].

The present study had some limitations [72]. The sample size, while adequate for preference ranking [52], was relatively small for modelling broader consumer behaviour, which may limit the generalisability of the regression findings. In addition, the participant cohort was relatively young (mean age 28 years) and included a higher proportion of individuals with a food‐related background than the general population, which limits the direct extrapolation of these findings to wider consumer groups in Ireland or Western markets. Protein content was estimated from ingredient labels rather than analytically measured, which may slightly overstate true protein values due to the presence of chitin‐derived non‐protein nitrogen in cricket‐based ingredients. Future studies should incorporate analytical confirmation, such as amino acid profiling, to provide more accurate protein quantification for insect‐based products. While microbiological safety, lipid oxidation and shelf‐life were not within the scope of the present study, their absence limits the assessment of the commercial applicability of the developed products and should be addressed in future research. Furthermore, since the controlled environment under which the sensory evaluation sessions were conducted may not fully reflect how consumers engage with such products in everyday settings, future studies should complement laboratory‐based evaluations with in‐home or real‐world testing to capture more context‐driven consumer behaviour. However, this study represents the first experimental investigation in Ireland exploring beef–cricket hybrids, offering a foundation for understanding consumer responses to hybrid insect–beef products and offering practical directions for further research and product development.

## 5. Conclusions

This study examined the feasibility of partially replacing beef in burgers with insects and its impact on physicochemical, techno‐functional and sensory properties, as well as consumer acceptance. Incorporating a hydrated cricket flour mixture yielded hybrid burgers with characteristics (colour, texture and moisture content and cooking loss) comparable to the beef‐only control. However, this functional equivalence did not ensure similar consumer acceptance across all formulations. Moderate substitution levels (up to 20% beef reduction) maintained comparable preference to the control, whereas higher substitutions (≥ 30%) led to a significant decline in preference, suggesting a threshold for acceptance. As qualitative feedback revealed, higher replacements were criticised for being inferior in sensory attributes, particularly taste and texture. Future studies could extend the current work by applying descriptive sensory methods (e.g., CATA or profiling) to characterise the sensory changes driving this decline in acceptance.

Acceptance was influenced by both consumer‐ and product‐related factors. Older participants, individuals with prior insect consumption experience, and those with a positive attitude towards food innovation were more open to purchasing hybrid burgers. Regression analysis showed that environmental impact, cost and protein content significantly predicted willingness to buy, explaining 31% of its variance. Environmental impact and cost exerted strong negative effects, while protein content had a modest positive influence, indicating that environmental benefits must align with favourable cost–value perceptions to drive adoption.

Overall, beef–cricket hybrid burgers showed promise as a strategy for partial meat replacement. However, the regression results highlighted that broader adoption would depend not only on sensory appeal but also on the perceived environmental and economic value of the product. Communication strategies that emphasise environmental benefits, nutritional value and palatable formulations will be critical to broader adoption of hybrid forms of insect‐based foods. To support the integration of insect protein into Western diets, future research should focus on optimising formulations for improved sensory appeal, understanding long‐term consumer behaviour, and targeting a broader demographic, especially regular meat consumers. Gradual introduction of insect ingredients in familiar food formats, as demonstrated in this study, represents a practical pathway towards more sustainable dietary transitions.

## Funding

This study is part of a PhD project funded by the Connaught Ulster Alliance Bursary (grant number PCUAB024).

## Conflicts of Interest

The authors declare no conflicts of interest.

## Supporting Information

These include Table S1 that presents the preliminary hedonic liking scores (mean ± SD; 5‐point scale) for beef insect hybrid burger formulations evaluated during product screening (*n* = 6).

## Supporting information


**Supporting Information** Additional supporting information can be found online in the Supporting Information section.

## Data Availability

The data that support the findings of this study are available on request from the corresponding author. The data are not publicly available due to privacy or ethical restrictions.
