# Supplementary material for: Product Development and Consumer Evaluation of Beef–Cricket Hybrid Burgers
Source: Int J Food Sci. 2026 Apr 22;2026:5598812. doi: 10.1155/ijfo/5598812 (PMC13100810; doi:10.1155/ijfo/5598812)
Supplement: Supplementary file 1 — Supporting Information Additional supporting information can be found online in the Supporting Information section. [file IJFO-2026-5598812-s001.docx]

**Supplementary Material**

**Table S1**. Preliminary hedonic liking scores (mean ± SD; 5-point scale) for beef insect hybrid burger formulations evaluated during product screening (n = 6) *.

| **Sample** | **Overall liking** | **Appearance liking** | **Flavour liking** | **Texture liking** |
| --- | --- | --- | --- | --- |
| Control | 4.3 ± 0.8 | 4.3 ± 0.8 | 3.8 ± 1.5 | 3.7 ± 1.4 |
| 5% beef replacement | 4.8 ± 0.4 | 4.8 ± 0.4 | 4.8 ± 0.4 | 4.8 ± 0.4 |
| 10% beef replacement | 4.3 ± 0.8 | 4.3 ± 1.0 | 4.3 ± 0.8 | 4.3 ± 1.0 |
| 15% beef replacement | 4.2 ± 1.2 | 4.2 ± 1.2 | 4.5 ± 0.5 | 4.0 ± 1.1 |
| 20% beef replacement | 4.2 ± 0.8 | 4.2 ± 1.3 | 4.4 ± 0.5 | 4.4 ± 0.9 |
| 30% beef replacement | 4.3 ± 0.5 | 4.5 ± 0.5 | 3.7 ± 1.0 | 4.2 ± 0.8 |
| 40% beef replacement | 4.0 ± 1.3 | 4.2 ± 1.2 | 3.5 ± 1.5 | 3.5 ± 1.4 |

*Scores were collected during preliminary product screening to inform sample selection (in addition to the group discussion afterwards). No inferential statistics were performed due to the small sample size.
